# Demographic, clinical and pathological characterisation of patients with colorectal and anal cancer followed between 2013 and 2016 at Maputo Central Hospital, Mozambique

**DOI:** 10.3332/ecancer.2021.1205

**Published:** 2021-03-16

**Authors:** Carlos Selemane, Luisa Jamisse, Jorge Arroz, Satish Túlsidas, António Gudo Morais, Carla Carrilho, Prassad Modcoicar, Moshin Sidat, Jessica Rodrigues, Daniel Moreira-Gonçalves, Mamudo Ismail, Lúcio Lara Santos

**Affiliations:** 1 Surgical Department, Maputo Central Hospital, 1653 Av Eduardo Mondlane, Maputo, Mozambique; 2 Pathology Department, Maputo Central Hospital, 1653 Av Eduardo Mondlane, Maputo, Mozambique; 3 Save the Children, P.O.Box 1854, Rua de Tchamba nº 398, Maputo, Mozambique; 4 Medical Oncology Department, Maputo Central Hospital, 1653 Av Eduardo Mondlane, Maputo, Mozambique; 5 Radiotherapy Service, Maputo Central Hospital, 1653 Av Eduardo Mondlane, Maputo, Mozambique; 6 Department of Pathology, Faculty of Medicine, Eduardo Mondlane University, 3453 Avenida Julius Nyerere, Maputo, Moçambique; 7 Gastroenterology Department, Maputo Central Hospital, 1653 Av Eduardo Mondlane, Maputo, Mozambique; 8 Department of Community Health, University of Eduardo Mondlane, 1653 Av Eduardo Mondlane, Maputo, Mozambique; 9 Global Health and Tropical Medicine, Institute of Hygiene and Tropical Medicine, Nova University of Lisbon,R. da Junqueira 100, 1349-008 Lisboa, Portugal; 10 Epidemiology Service, Portuguese Institute of oncology, Rua Dr. António Bernardino de Almeida 4200-072, Porto, Portugal; 11 Research Center in Physical Activity, Health and Leisure (CIAFEL), Faculty of Sport, University of Porto, R. Dr. Plácido da Costa 91, 4200-450, Porto, Portugal; 12 Experimental Pathology and Therapeutics Research Group, Portuguese Institute of oncology, Rua Dr. António Bernardino de Almeida 4200-072, Porto, Portugal; 13 Surgical Oncology Department, Portuguese Institute of oncology, Rua Dr. António Bernardino de Almeida 4200-072, Porto, Portugal; 14 ONCOCIR-Education and Care in Oncology, Lusophone , Africa, Rua de Quires 168-10J, Moreira da Maia , Portugal

**Keywords:** Africa, Mozambique, colorectal cancer, anal cancer

## Abstract

**Purpose:**

The aim of this study was to investigate colorectal cancer (CRC) data and anal cancer data from Maputo Central Hospital (MCH), the largest hospital and a reference for oncological diseases in Mozambique, with the aim of characterising the disease profile in view to define an appropriate control programme.

**Methods:**

MCH records from the Pathology and Surgery Services and MCH Cancer Registry database were assessed to obtain retrospective clinical and pathologic data of patients with CRC or anal cancer admitted to and treated between 13 December 2013 and 23 March 2016.

**Results:**

The female gender was more prevalent (54.8%), even when anal cancers were excluded. Median age was 54 years (20–99). Most patients (51.6%) lived in the city of Maputo. The most common presenting symptom was found to be rectal bleeding. Adenocarcinoma was the most frequent histological type, and the most prevalent anatomical site was the rectum. Most of the cases were diagnosed at MCH in advanced stages. Colostomy was the most frequent surgical procedure and performed in 38.7% of the patients. Most cases of anal cancer occurred in human immunodeficiency virus-infected patients. Most patients had a poor prognosis due to advanced stage at first diagnosis.

**Conclusion:**

We observed an increase in cases of CRC and anal cancer in Mozambique and mostly diagnosed at advanced stages, which anticipates a dismal prognosis. Our data supports the urgent need of a comprehensive public health programme dedicated to solving this growing concern.

## Background

Colorectal cancer (CRC) is the third most deadly type of cancer worldwide, with approximately 881,000 estimated deaths in 2018, but data concerning cancer rates in Sub-Saharan Africa (SSA) is extremely poor [1]. The crude incidence rate of CRC in SSA was estimated as 4.0/100,000 (4.4/100,000 for men and 3.7/100,000 for women) and the incidence increased with age with the highest rates particularly reported for South Africa [2]. The population-based cancer registry of Mozambique, with data from the city of Beira and the city of Maputo, revealed that CRC has low age-standardised incident rates (ASIR) (Table 1) [3]. However, in terms of the variation in Maputo, the ASIR between 1956–1961 and 2015–2016 was at least 40% higher for CRC (66.7% in males and 40.0% in females) [4]. Over the last 30 years in West Africa, published evidence has shown decade-by-decade increases in the incidence of CRC [5]. Parker *et al* [6] recently underlined the increased incidence of CRC in rural Kenya. The rates of cancer (including CRC) are dramatically increasing partly because of the ageing of the population, and partly due to the rapid ‘globalisation’ and the adoption of the associated risk factors within these populations [7]. These risk factors include physical inactivity, smoking and alcohol consumption and poor nutrition [8]. On the other hand, the increase of the registered cases can also reflect improvements in diagnostic measures as well as in the quality of clinical registries, which ultimately allows a more accurate measure of the cases of CRC [9]. In SSA, CRC frequently occurs at an earlier age, often with distinctive histological characteristics [9]. The increase in the incidence rate of this malignancy implies that we must anticipate actions, soon, holding potential to mitigate deaths. On the other hand, anal cancer is rare, comprising ~2.7% of all malignancies of the digestive system [10]. According to Zuma *et al* [11], the prevalence of anal squamous cell carcinoma is much higher in individuals with human immunodeficiency virus (HIV) infection. HIV-positive patients present at a younger age and with locally advanced disease are less responsive to standard treatment, and their survival is poorer [11]. However, clinical evaluation and differential diagnosis between advanced malignancies of the lower rectum and the anal cancer are difficult to carry out fundamentally in a context where malignant tumours are diagnosed late. Thus, this study reviews CRC data and anal cancer data from the Maputo Central Hospital (MCH), the largest hospital and a reference for oncological diseases in Mozambique, with aim of characterising the disease profile in view to define an appropriate control programme.

## Methods

### Study setting

The study was carried out at MCH, a quaternary referral hospital in Maputo, the Capital City of Mozambique.

### Study design and data collection

MCH records were assessed from the Pathology and Surgery Services and MCH Cancer Registry database to obtain retrospective clinical and pathologic data of patients with CRC or anal cancer admitted to and treated at MCH between 13 December 2013 and 23 March 2016. The following patient characteristics were captured: demographics, clinical presentation, tumour location and histopathological findings. Age at presentation, family history of cancer, place of residence and diagnosis, American Joint Committee on Cancer staging, treatments performed for each patient (surgery and chemotherapy) and follow-up data were also obtained from the MCH records [12].

### Data management and analysis

The data were introduced into Microsoft Excel® and data analysis was conducted using SPSS 21®. Continuous variables are presented as median (Min and Max) and categorical variables as frequencies and percentages. Chi-squared or Fisher’s exact tests were used to evaluate the association between two categorical variables. Comparisons between groups were performed, using independent samples *t*-tests (or Mann–Whitney) and Analysis of variance (ANOVA) (or Kruskal–Wallis) tests for continuous variables as appropriate.

### Ethical considerations

The study was based on Cancer Registry of the MCH and was approved by the Joint Institutional Bioethics Committee of the Faculty of Medicine, Eduardo Mondlane University and MCH (number – CIBS FM&HCM/71/2017).

## Results

**Figure 1.**
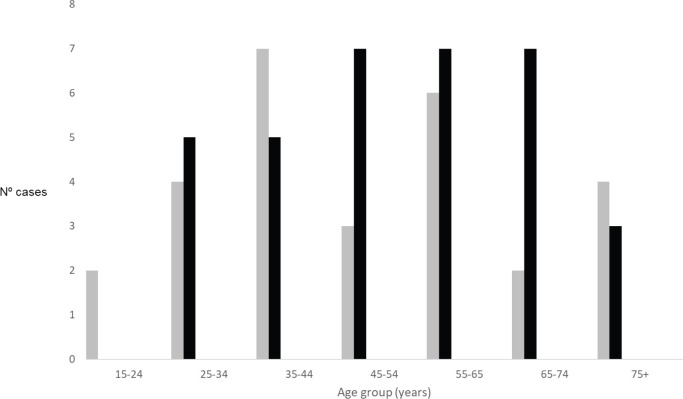
Number of cases by age group and gender (grey – male; black – female).

**Figure 2.**
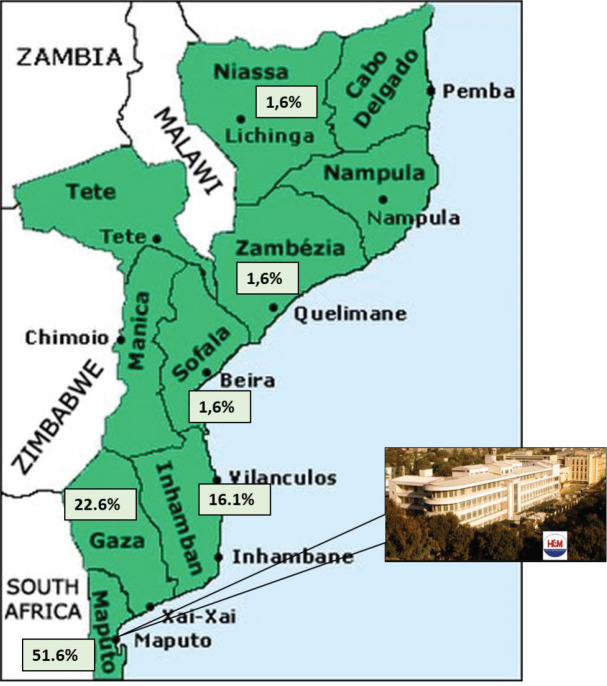
Percentage of cases in the series by province and MCH.

Demographics, clinical and pathological characteristics of MCH series are summarised in Table 2. Sixty-two patients were admitted and treated consecutively at MCH from 2013 to 2016. Median age was 54 years (20–99). The overall incidence of cancer increased with age (Figure 1). The female gender was more prevalent (54.8%), even if cancers of the anus were excluded. Woman accounted for 70% of all malignant tumours of the anus. All patients were black. Most patients (51.6%) lived in the city of Maputo or in the neighbouring provinces (Figure 2). Thirty-two patients (50.8%) were admitted to the emergency room, one patient was transferred from another hospital (1.6%) and the remaining patients (47.6%) were admitted after an outpatient surgery consultation. No cases reported any known family history of cancer. The most frequent reason for hospital admission was rectal bleeding (38.1%). In 27 cases (43.5%), the diagnosis was made by colonoscopy and the rest mainly by clinical evaluation. In 20 patients (32.3%), the neoplasm was considered clinically malignant and no biopsy was performed due to the advanced stage of the disease. In 42 patients for whom histology was performed, adenocarcinoma was the most frequent histological type (67.8%), distributed as follows: rectal cancer 20 (64.5%), colon cancer 7 (22.5%) and anal cancer 2 (6.5%). Twenty-four patients (38.7%) were assessed for HIV profile and only 7 (11.3%) were positive. Of these positive cases, the tumour was in the anus (5 patients) and rectum (2 patients). Of the 10 squamous cell carcinomas, 7 were found in the anus, 3 in the lower rectum. At the time of diagnosis, the disease was found at advanced stage in most patients (Table 2). This fact determined that a great proportion of patients only received palliative treatment (35.5%). Colostomy was the most frequent surgical procedure and performed in 38.7% of the patients. Eleven (17.7%) patients in the series were treated surgically with curative intent (5 colon, 5 rectum and 1 anal cancer), and 5 of these patients also received adjuvant chemotherapy. In the series, only four (6.5%) patients received palliative chemotherapy (in CRC, the FOLFOX protocol is used since Irinotecan is not always available; in the anal cancer, the most used protocol combines Fluorouracil (5-FU) and Cisplatin). Although radiotherapy is now available in MCH, these series of patients did not benefit from this therapeutic option since at that time the system was being assembled. The loss to follow-up rate was 75.8% (47 patients, including some patients treated with curative intent) and the reason was mainly due to loss of tracking (e.g. telephone contact was no longer available and/or did not attend the appointments). We calculated the overall survival (from the date of diagnosis to the date of death by cancer) in the remaining 15 patients of this series, being rectal cancer 10 (66.7%) and colon cancer 5 (33.3%), thus the median survival was 6.8 months (varying from 1 month to 27.6 months).

## Discussion

This longitudinal retrospective study aimed to characterise the demographic, clinical and pathological profile of patients with CRC and anal cancer at MCH, in a series of consecutive patients.

We found in this series of patients that cancer is relatively more common among the female gender, even when cancers of the anus are excluded, though this difference was not significant. Data from the population-based cancer registry of Maputo (2015−2017) and Beira (2014−2017) revealed that the number of cases is higher in males when malignant tumours of the anus are not included [3]. The largest systematic review published so far on CRC in SSA also corroborates the higher incidence in males than females (4.38 for men and 3.69 for women), as well as increased incidence with ageing [2]. Regarding age, we observed in our series and in the data of the cancer registry of Mozambique mentioned above, that the incidence also increases with age (CRC and anal cancer). McCabe *et al* [13] found that black South African (SA) patients with CRC present at a significantly younger age in comparison to other SA race groups, suggesting need to assess in the future whether there are cases with family aggregation and co-related genetic alterations. Despite the relatively young age of some patients in our series, we did not find any reference to the family history of cancer in our series of cases. However, it will be necessary to pay more attention to this aspect in the future to understand if there are cases with family aggregation or genetic alterations that suggest the possibility of cases of hereditary CRC.

The percentage of cases in different anatomical locations varies between publications, but most of them corroborate our observation that rectum is the most common site [2, 14–15]. The histological diagnostic confirmation rate, in the series, was 67.7%, and as expected adenocarcinoma was the most frequent tumour in the colon and rectum and squamous cell carcinoma the most frequent in the anus [16]. The relatively low rate of histological confirmation occurred because a considerable number of patients were not biopsied because they presented with an advanced stage of the disease at the time of diagnosis, with extremely poor performance status and needed the best supportive care. Similar situations occurred in other African countries, where a high number of patients are not deemed fit for treatment at the time of diagnosis [17]. In Africa, cancer of the anus is associated with HIV [9] and we also noted a higher prevalence of HIV in these patients in our series. In the future, it will be essential to study the prevalence of Human papillomavirus (HPV) infections in these patients as it was done in Zimbabwe given the potential association of the co-infection of these two viruses in these patients [18]. The quadrivalent HPV vaccine has been demonstrated to prevent vaccine-associated persistent anal HPV infections as well as anal intraepithelial neoplasia grades 2–3 and the HPV types detected in anal cancer are included in the 9-valent vaccine. Thus, the 9-valent HPV vaccine, when administered to boys and girls prior to the onset of sexual activity, should effectively prevent anal cancer [19]. In countries in which >50% of girls and young women have received HPV vaccination, there has been a significant decrease in anogenital warts in males aged <20 years [20].

The most common presenting symptom was found to be ‘rectal bleeding’ (even if cancers of the anus are excluded), which differs from most studies in which the most common presenting symptom was found to be the ‘bloody stool’ [2]. We attribute this observation to the fact that most of the patients in our series were diagnosed at an advanced stage. Indeed, ‘rectal bleeding’ appears to be one of the most common signs associated with the more advanced stages of the disease [14, 21]. On the other hand, ‘rectal bleeding’ and ‘bloody stool’ are often confounded, which could also be the case. Late diagnosis is a major issue in SSA. In 300 consecutive patients that were enrolled in a recent Nigerian study, most of them were also at an advanced stage with distant metastases [17]. It is widely believed that the incidence of CRC in SSA countries still does not justify the establishment of organised screening. The Nigerian National Cancer Strategy (2018–2022) has identified CRC screening as a priority and endorses the establishment of a national screening programme, but it has been a source of controversy [22]. Our concern is that we do not have an adequate structure in Mozambique to care for patients with CRC and anal cancer who currently need our medical services. A screening programme, even inexpensive, using, for example, Fecal occult blood test (FOBT) and fecal immunochemical test (FIT) would create exhaustion of services and have no impact. Despite the liable survival estimate, the stage of the disease found in the series of patients described in this study anticipates limited survival and high mortality.

According to data from MCH cancer registry, cases of CRC and anus are increasing, as it is happening across many sub-Saharan African countries [23]. Therefore, it is mandatory to create conditions for an earlier diagnosis and treatment of this deadly disease. This will require alerting and educating the population and health professionals about the disease and providing hospitals at the tertiary level with adequate early diagnostic and effective therapeutic resources. In this sense, the missing technical resources were identified, the health personnel’s knowledge about the CRC was evaluated and specific training was organised in order to fill the gaps [24]. It will be important to establish multidisciplinary training programmes dedicated to the treatment and monitoring of patients with CRC and anal cancers. The application of the recently approved National Curriculum to Advance Surgical Oncology in Mozambique will be an important step [25]. A high proportion of people with CRC are young and some have pathological and molecular features suggestive of hereditary cancer [9]. The identification and the investigation of hereditary factors is an expensive activity. However, it must be implemented because it saves lives and can count on the support of international consortia [26]. In addition, it will be crucial to organise the follow-up of these patients, as it is being organised for breast cancer in other countries in Africa, to reduce losses from follow-up, ensure timely treatment of recurrences, systemic and palliative treatment, ultimately promoting quality of life for these patients [27]. The activities that we consider essential to change the current CRC and anal cancer picture in Mozambique are condensed in the programme that we propose (Table 3).

## Limitations

This study has the limitation of using secondary retrospective data, with missing values and a high rate of lost to follow-up so, the survival data is anecdotal. The low histological confirmation rate that we had is a frequent feature in SSA and should be changed quickly [28]. However, available demographic, clinical and pathological data allowed characterising the disease in context of referral hospital in Mozambique and elaborating objective suggestions for improvements.

## Conclusions

In context of a substantial increase in cases of CRC and anal cancer in Mozambique, much needs to be done for prevention, earlier diagnosis and improvement of survival. A comprehensive public health programme is needed as well as appropriate diagnostic and therapeutic resources allocation in quaternary hospitals dedicated to this pathology required within Mozambican Health System.

### What is known about this topic?

CRC is already the third leading cause of cancer related deaths in the world, and its incidence is steadily rising in developing nations;

The ASIR in low-human development index (HDI) nations is higher in men than in women (8.4/100,000 versus 5.9/100,000, respectively);

In SSA, the incidence of CRC is increasing in both genders;

Anal cancer is on the rise in SSA.

### What this study adds

This is the first study describing the patients’ characteristics in CRC and anal cancers in Mozambique;

Most cases in this study were diagnosed in advanced stages, to whom palliative care was the only possible treatment;

Adenocarcinoma was the most frequent histological type, and the most prevalent anatomical site was the rectum;

Most cancers of the anus were among HIV infected patients.

## Conflicts of interest

The authors declare that they have no conflict of interest.

## Funding statement

The authors declare that they had no financial support for this work.

## Authors' contributions

This study was conceptualised, designed and written by CS, MI and LLS. Acquisition of data was carried out by CS, LJ, ST, CC, PM, AGM and MI. Analysis and interpretation of data were done by JA, JR, DMG and LLS. CS, MS, JR, DMG, MI and LLS drafted and revised the article for important intellectual content. All authors read and agreed to the final version of this manuscript.

## Figures and Tables

**Table 1.** CRC and anus cancer incidence in Mozambique (data from population-based cancer registry) adapted from Lorenzoni et al [3].

	Maputo 2015–2017			Beira 2014–2017		
Gender	Cases	ASIR	MV	Cases	ASIR	MV
Male						
Colorectal	32	2.0	78%	11	2.0	64%
Anus	2	0.3	50%	2	0.3	0%
Female						
Colorectal	29	2.6	90%	9	1.8	67%
Anus	11	0.9	91%	7	1.0	86%

ASIR, Age-standardised incident rates (World); MV, Microscopic validation

**Table 2.** Demographics, clinical and pathological characteristics of MCH series.

Variables	Total (*n* = 62)	Male (*n* = 28)	Female (*n* = 34)	*p*
**Age (years)** Median (Min; Max)	54 (20–99)	51.5 (20–99)	55.0 (29–78)	ns
**Residence (%)** Maputo Gaza Inhambane Niassa Sofala Zambezia Unknown	32 (51.6) 14 (22.6) 10 (16.1) 1 (1.6) 1 (1.6) 1 (1.6) 3 (4.8)	16 (57.1) 4 (14.3) 5 (17.9) 1 (3.6) 0 (0.0) 0 (0.0) 2 (7.1)	16 (47.1) 10 (29.4) 5 (14.7) 0 (0.0) 1 (2.9) 1 (2.9) 1 (2.9)	ns
**Symptoms (%)** Loss of weight Abdominal pain Anaemia Tenesmus Intestinal occlusion Rectal bleeding Blood in stool Rectovaginal fistula Tumour (clinic or endoscopic)	5 (8.1) 1 (1.6) 2(3.2) 2 (3.2) 11 (17.7) 24 (38.7) 2 (3.2) 1 (1.6) 14 (22.6)	3 (10.7) 0 (0.0) 1 (3.6) 0 (0.0) 5 (17.9) 11 (39.3) 1 (3.6) 0 (0.0) 6 (21.4)	2 (5.9) 1 (2.9) 1 (2.9) 1 (2.9) 6 (17.6) 13 (38.2) 1 (2.9) 1 (2.9) 8 (23.5)	ns
**Anatomic site (%)** Caecum Ascending colon Left colic flexure Descending colon Sigmoid colon Rectosigmoid Rectum Anus Unknown	3 (4.8) 5 (8.1) 1 (1.6) 2 (3.2) 1 (1.6) 1 (1.6) 36 (58.1) 10 (16.1) 3 (4.8)	2 (7.1) 3 (10.7) 1 (3.6) 0 (0) 1 (3.6) 0 (0) 16 (57.1) 3 (10.7) 2 (7.1)	1(2.9) 2 (5.9) 0 (0) 2 (5.9) 0 (0) 1 (2.9) 20 (58.8) 7 (20.6) 1 (2.9)	ns
**Histology (%)** Adenocarcinoma Squamous cell carcinoma Carcinoid No histology	31 (50.0) 10 (16.1) 1 (1.6) 20 (32.3)	15 (53.6) 3 (10.7) 0 (0.0) 10 (35.7)	16 (47.1) 7 (20.6) 1 (2.9) 10 (29.4)	ns
**Stage (%)** II III IV Unknown	5 (8.1) 19 (30.6) 16 (25.8) 22 (35.5)	2 (7.1) 9 (32.1) 6 (21.4) 11 (39.3)	3 (8.8) 10 (29.4) 10 (29.4) 11 (32.4)	ns
**Surgical procedure (%)** Excisional Biopsy Colostomy Hartmann operation Right colectomy Left colectomy Sigmoidectomy Low anterior resection Abdominoperineal resection Laparotomy Palliative care (without surgery)	1 (1.6) 24 (38.7) 3 (4.8) 3 (4.8) 1 (1.6) 1 (1.6) 1(1.6) 4 (6.5) 2 (3.2) 22 (35.5)	0 (0.0) 7 (25.0) 1 (3.6) 2 (7.1) 1 (3.6) 1(3.6) 0 (0.0) 3 (10.7) 1 (3.6) 12 (42.9)	1 (2.9) 17 (50.0) 2 (5.9) 1 (2.9) 0 (0.0) 0(0.0) 1 (2.9) 1 (2.9) 1 (2.9) 10 (29.4)	ns

ns, Not significant

**Table 3.** Actions needed for CRC programme in Mozambique.

Task	Actions
Structural developments	Acquire gastroenterology, operating room resources and create the comprehensive CRC dedicated unit (medical oncology, surgical oncology, radio-oncology, dedicate nurses, pharmacists, and psychologists, geneticists). To promote the reports standardisation of pathology, stage, surgical procedure, radiation and medical oncology treatment and follow-up.
Capacity building and awareness	Promote an educational programme that includes doctors, nurses and schoolteachers.
Prevention and diagnosis	Create a proactive programme for early detection and hereditary cancer consultation. HPV vaccination. Improve the use and accuracy of pathology diagnosis (including Microsatellite instability (MSI) status). Make the assessment of crucial genetic mutations available.
Treatment decision	Multidisciplinary tumour board
Adequate treatment	Surgery: The best surgical treatment (surgical oncology training); Systemic therapy: drugs availability, affordability and uninterrupted supply, safe storage and preparation, adequate prescription and administration, management of side effects. Radiation therapy: to be included in CRC treatment protocol, management of side effects related to radiation, including palliative radiotherapy. Accessibility to cost-effective chemotherapy or radiation therapy, and palliative care (including palliative surgery).
Follow-up	Shared and supported follow-up programme including all levels of care and adequate registration of follow-up data (include primary care nurses and telehealth resources).
Palliative care	Pain control and adequate management of end-of-life care.

## References

[1] Rawla P, Sunkara T, Barsouk A Epidemiology of colorectal cancer: incidence, mortality, survival, and risk factors. Prz Gastroenterol.

[2] Graham A, Adeloye D, Grant L (2012). Estimating the incidence of colorectal cancer in Sub-Saharan Africa: a systematic analysis. J Glob Health.

[3] Lorenzoni CF, Ferro J, Carrilho C (2020). Cancer in Mozambique: results from two population-based cancer registries. Int J Cancer.

[4] Carrilho C, Fontes F, Tulsidás S (2019). Cancer incidence in Mozambique in 2015–2016: data from the Maputo Central Hospital Cancer Registry. Eur J Cancer Prev.

[5] Irabor DO (2017). Emergence of colorectal cancer in West Africa: accepting the inevitable. Niger Med J.

[6] Parker RK, Ranketi SS, McNelly C (2019). Colorectal cancer is increasing in rural Kenya: challenges and perspectives. Gastrointest Endosc.

[7] Negin J, Cumming R, de Ramirez SS (2011). Risk factors for non-communicable diseases among older adults in rural Africa. Trop Med Int Health.

[8] Jemal A, Bray F, Center MM (2011). Global cancer statistics. CA Cancer J Clin.

[9] Katsidzira L, Gangaidzo I, Thomson S (2017). The shifting epidemiology of colorectal cancer in sub-Saharan Africa. Lancet Gastroenterol Hepatol.

[10] Siegel RL, Miller KD, Jemal A (2019). Cancer statistics. CA Cancer J Clin.

[11] Zuma NP, Ngidi S, Madiba TE (2020). Anal squamous cell carcinoma in KwaZulu-Natal Province, South Africa, with special reference to the influence of HIV infection on clinical presentation and management outcome. SAMJ: South Afr Med J.

[12] Edge SB, Compton CC (2010). The American Joint Committee on Cancer: the 7th edition of the AJCC cancer staging manual and the future of TNM. Ann Surg Oncol.

[13] McCabe M, Perner Y, Magobo R (2019). Descriptive epidemiological study of South African colorectal cancer patients at a Johannesburg Hospital Academic institution. JGH Open.

[14] Yawe KT, Bakari AA, Pindiga UH (2007). Clinicopathological pattern and challenges in the management of colorectal cancer in Sub–Saharan Africa. J Chinese Clin Med.

[15] Gatta G, Ciccolallo L, Capocaccia R (2003). Differences in colorectal cancer survival between European and US populations: the importance of sub-site and morphology. Eur J Cancer.

[16] Abdulkareem FB, Abudu EK, Awolola NA (2008). Colorectal carcinoma in Lagos and Sagamu, Southwest Nigeria: a histopathological review. World J Gastroenterol.

[17] Sharma A, Alatise OI, Adisa AO (2019). Treatment of colorectal cancer in Sub-Saharan Africa: Results from a prospective Nigerian hospital registry. J Surg Oncol.

[18] Chinyowa S, Palefsky JM, Chirenje ZM (2018). Anal human papillomavirus infection in HIV-positive men and women at two opportunistic infections clinics in Harare, Zimbabwe. BMC Public Health.

[19] Stier EA, Chigurupati NL, Fung L (2016). Prophylactic HPV vaccination and anal cancer. Hum Vaccin Immunother.

[20] Drolet M, Benard E, Boily M (2015). Population-level impact andherd effects following human papillomavirus vaccination pro-grammes: a systematic review and meta-analysis. Lancet Infect Dis.

[21] Chalya PL, Mchembe MD, Mabula JB (2013). Clinicopathological patterns and challenges of management of colorectal cancer in a resource-limited setting: a Tanzanian experience. World J Surg Oncol.

[22] Knapp GC, Alatise OI, Olasehinde OO (2019). Is colorectal cancer screening appropriate in Nigeria?. J Glob Oncol.

[23] Ntombela XH, Sartorius B, Madiba TE (2015). The clinicopathologic spectrum of anal cancer in KwaZulu-Natal Province, South Africa: analysis of a provincial database. Cancer Epidemiol.

[24] Atílio M, Matchecane C, Adriano T (2018). Identifying barriers and finding solutions to implement best practices for cancer surgery at Maputo Central Hospital, Mozambique. Ecancermedicalscience.

[25] Morais A, Simão M, Cossa M (2021). Designing a national curriculum to advance surgical oncology in Mozambique: a Delphi Consensus Study. J Surg Educ.

[26] Ashktorab H, Ahuja S, Kannan L (2016). A meta-analysis of MSI frequency and race in colorectal cancer. Oncotarget.

[27] McKenzie F, Zietsman A, Galukande M (2016). African Breast Cancer-Disparities in Outcomes (ABC-DO): protocol of a multicountry mobile health prospective study of breast cancer survival in sub-Saharan Africa. BMJ Open.

[28] Fleming K (2019). Pathology and cancer in Africa. Ecancermedicalscience.

